# Severe Babesiosis With Multifactorial Hemolysis and Pulmonary Complications in an Asplenic Patient at a Tertiary Cancer Center

**DOI:** 10.7759/cureus.94343

**Published:** 2025-10-11

**Authors:** Gemechu Ayana, Scotty Carlson, Baziliya K Keraga, Goitom Weldearegay, Steven Maron

**Affiliations:** 1 Internal Medicine, State University of New York (SUNY) Downstate Health Sciences University, New York City, USA; 2 Internal Medicine, Weill Cornell Medicine, Cornell University, New York City, USA; 3 Internal Medicine, Jimma University, Jimma, ETH; 4 Oncology, Memorial Sloan Kettering Cancer Center, New York City, USA

**Keywords:** antibiotic allergy, asplenia, babesiosis, memorial sloan kettering, pancreatic cancer

## Abstract

Babesiosis is a tick-borne protozoal infection that can cause life-threatening disease in asplenic or immunocompromised patients. We report a 71-year-old woman with prior distal pancreatectomy and splenectomy who developed severe babesiosis complicated by pulmonary involvement and complement-mediated hemolysis. Initial therapy with clindamycin-atovaquone and partial exchange transfusion was required due to a reported azithromycin allergy; following successful rechallenge, she tolerated guideline-based azithromycin-atovaquone. Despite parasite clearance, she developed complement-mediated hemolysis confirmed by a complement C3-positive, IgG-negative direct antiglobulin test.

A 71-year-old woman with a history of distal pancreatectomy and splenectomy for pancreatic carcinoma (without recurrence) presented from upstate New York with one week of fever, chills, headache, fatigue, and myalgias. She denied a known tick bite but reported a small pink skin lesion. Initial laboratory evaluation revealed thrombocytopenia, transaminitis, and hyperbilirubinemia; hepatitis, Epstein-Barr virus, and cytomegalovirus testing were negative, while *Babesia microti* polymerase chain reaction (PCR) was positive. Because of a reported azithromycin allergy manifesting as hives, she was started on clindamycin-atovaquone. On admission to Memorial Sloan Kettering Cancer Center, her hemoglobin was 7.6 g/dL, lactate dehydrogenase (LDH) 392 U/L, and parasitemia 9.7%. Partial exchange transfusion (four of nine units) lowered parasitemia to 5.2%. The clinical course was complicated by recurrent fevers, hypotension, and hypoxia requiring a high-flow nasal cannula; chest CT demonstrated bilateral ground-glass opacities and pleural effusions. On hospital day 10, an azithromycin challenge was tolerated, and therapy was transitioned to azithromycin-atovaquone, with parasitemia clearing by day 16. Despite clearance of parasitemia, the patient developed worsening hemolysis characterized by a complement C3-positive, immunoglobulin (IgG)-negative direct antiglobulin test, consistent with complement-mediated hemolysis; cold agglutinin titer was negative. She experienced a hemoglobin nadir of 6.5 g/dL, lactated dehydrogenase peaking at 1,573 U/L, haptoglobin <8 mg/dL, and mild transient elevations in liver enzymes. She required multiple transfusions, though immunosuppressive therapy was avoided, and hematologic parameters improved as the infection resolved. Echocardiography revealed preserved systolic and diastolic function with a left ventricular ejection fraction of 68%. This case highlights several important aspects of severe babesiosis. First, asplenic patients represent a high-risk population, prone to higher parasitemia and serious complications. Second, therapeutic challenges may arise in the setting of drug allergies, but careful reevaluation can allow the use of guideline-based azithromycin-atovaquone therapy. Third, this case underscores the emerging recognition of complement-mediated autoimmune hemolysis in babesiosis, demonstrated by worsening hemolysis despite parasite clearance and a complement C3-positive, immunoglobulin G (IgG)-negative direct antiglobulin test. Finally, optimal outcomes required a multidisciplinary approach involving infectious disease, hematology, and transfusion medicine specialists, reflecting the complexity of management in severe and atypical babesiosis.

## Introduction

Babesiosis is a tick-borne parasitic infection caused primarily by *Babesia microti* in the United States and transmitted through the bite of the *Ixodes scapularis* tick, which also vectors *Borrelia burgdorferi* and *Anaplasma phagocytophilum*. The parasite infects red blood cells, leading to intra-vascular hemolysis, systemic inflammation, and, in severe cases, multiorgan dysfunction [1-3].

Although babesiosis may present as a mild and self-limited illness in immunocompetent individuals, it can cause severe disease in asplenic, elderly, or immunocompromised patients due to impaired clearance of parasitized erythrocytes and dysregulated immune responses. These high-risk populations often experience high parasitemia, prolonged illness, and serious complications, such as pulmonary involvement and immune-mediated hemolysis.

The incidence of babesiosis in the United States has been steadily increasing, with the highest rates observed in the Northeast and upper Midwest, particularly in New York, Massachusetts, Connecticut, and Rhode Island. This rising trend, combined with the growing population of immunocompromised and asplenic patients, underscores the complexity of management and the need for heightened clinical awareness. Furthermore, recent literature describes emerging complications, including complement- or immune-mediated hemolysis, which remain under-recognized and warrant further research [4-9].

We present a case of severe babesiosis in an asplenic patient complicated by pulmonary involvement and complement-mediated hemolysis, highlighting the importance of early recognition, allergy reassessment to ensure appropriate therapy, and multidisciplinary management guided by current Infectious Diseases Society of America (IDSA) recommendations.

## Case presentation

A 71-year-old woman with a history of distal pancreatectomy and splenectomy for pancreatic carcinoma (no recurrence) presented with one week of fever, chills, headache, fatigue, and myalgias while visiting upstate New York, an endemic region for *Babesia microti* [2,8-10]. She denied a known tick bite but noted a small pink lesion on her leg. Initial laboratory evaluation showed thrombocytopenia, transaminitis, and hyperbilirubinemia. Tests for hepatitis viruses, Epstein-Barr virus (EBV), and cytomegalovirus (CMV) were negative. Polymerase chain reaction (PCR) for *Babesia microti* was positive, confirming the diagnosis.

Due to a self-reported azithromycin allergy (manifested as hives), the patient was started on clindamycin plus atovaquone, an accepted alternative regimen for babesiosis [11,12]. According to the Infectious Diseases Society of America (IDSA) guidelines, the first-line therapy is atovaquone plus azithromycin, while clindamycin plus quinine is reserved for patients who cannot tolerate or do not respond to the preferred regimen [12]. In this case, due to limited quinine availability and the reported azithromycin allergy, clindamycin and atovaquone were initiated, given the severity of presentation.

**Figure 1:**
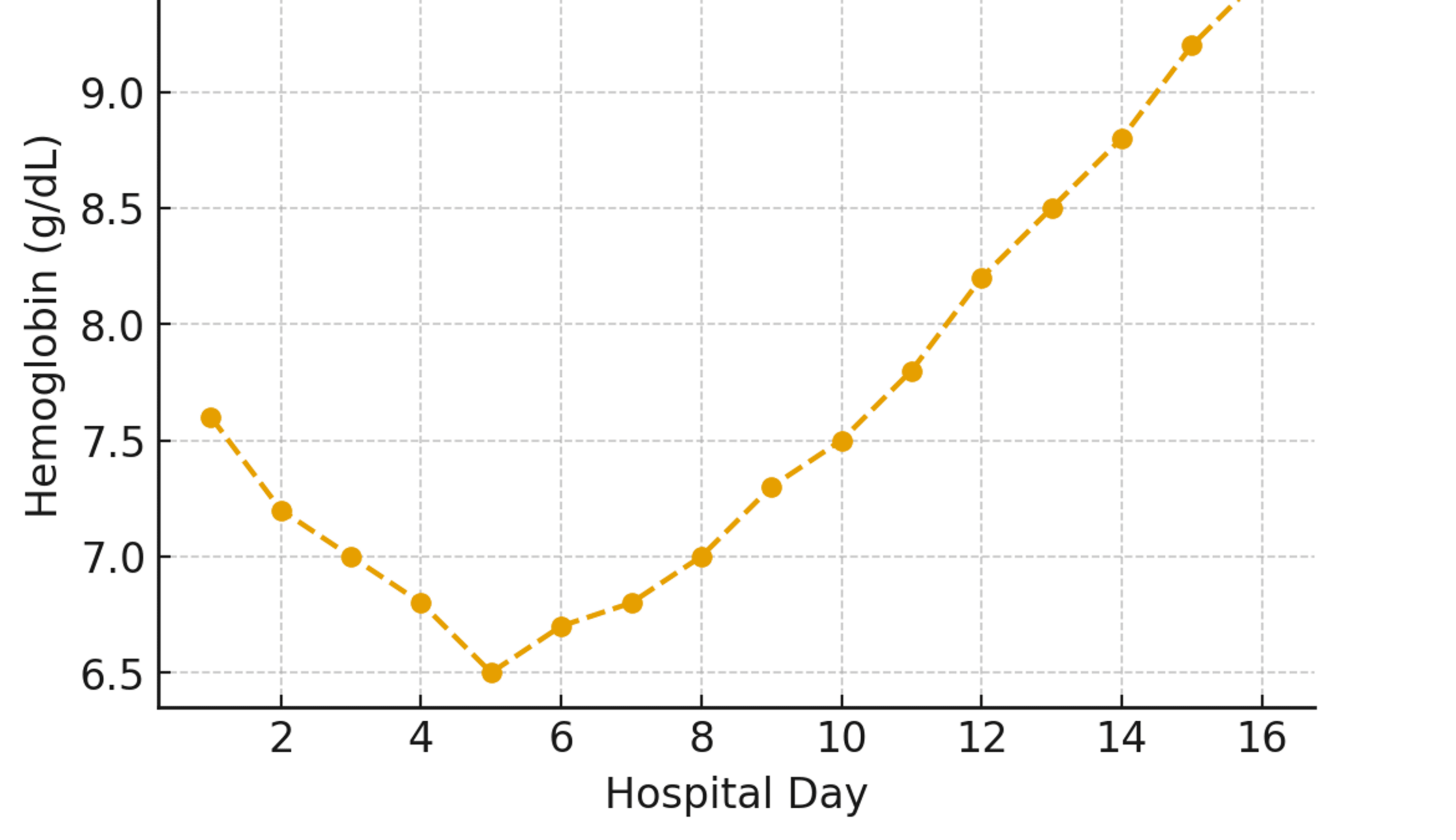
Trends of hemoglobin during hospitalization. Serial hemoglobin values during the 16-day hospitalization demonstrate an initial decline from 7.6 g/dL to a nadir of 6.5 g/dL by hospital day five, consistent with hemolytic anemia secondary to *Babesia microti* infection. Following initiation of combination therapy with atovaquone and clindamycin and later azithromycin, hemoglobin levels gradually improved, reaching 9.1 g/dL by discharge. Supportive management, including transfusion and intra-venous hydration, contributed to recovery. The trend illustrates the typical pattern of hemolysis and subsequent hematologic improvement following effective antiparasitic therapy.

**Figure 2:**
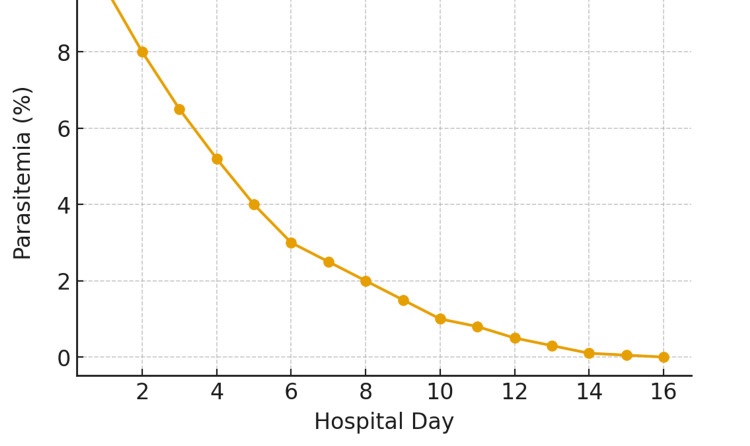
Parasitemia trend during hospitalization. Peripheral blood smear evaluations revealed a progressive decrease in parasitemia from approximately 9% on admission to <0.5% by hospital day 14, with complete clearance by day 15. Daily smear quantification was performed using Wright-Giemsa staining under oil immersion. The decline in parasitemia closely paralleled clinical improvement and hemoglobin recovery (as shown in Figure 1). The rapid parasitologic response underscores the efficacy of atovaquone plus azithromycin therapy in immunocompetent patients and highlights the importance of early recognition and treatment to prevent complications, such as hemolytic anemia and multiorgan dysfunction.

On admission to Memorial Sloan Kettering Cancer Center, her hemoglobin was 7.6 g/dL (Figure 1), lactate dehydrogenase (LDH) was 392 U/L (Table 1), and parasitemia was 9.7% (Figure 2). Partial exchange transfusion (four of nine units) reduced parasitemia to 5.2%, consistent with recommendations for severe babesiosis with high-level parasitemia [2,12]. During hospitalization, she developed recurrent fevers, hypotension, and hypoxia, requiring a high-flow nasal cannula. Chest imaging revealed bilateral ground-glass opacities and pleural effusions. On hospital day 10, an azithromycin challenge was successfully performed, allowing transition to the preferred azithromycin-atovaquone regimen, with clearance of parasitemia by day 16.

**Table 1:** Laboratory value trends during hospitalization. LDH: lactate dehydrogenase

Hospital day	Parasitemia (%)	Hemoglobin (g/dL)	LDH (U/L)
1	9.7	7.6	392
2	8	7.2	500
3	6.5	7	650
4	5.2	6.8	900
5	4	6.5	1,100
6	3	6.7	1,300
7	2.5	6.8	1,500
8	2	7	1,573
9	1.5	7.3	1,500
10	1	7.5	1,400
11	0.8	7.8	1,200
12	0.5	8.2	1,000
13	0.3	8.5	800
14	0.1	8.8	650
15	0.05	9.2	500
16	0	9.5	400

Despite parasite clearance, she developed worsening hemolysis characterized by a complement C3-positive, IgG-negative direct antiglobulin test (DAT), consistent with complement-mediated hemolysis, while the cold agglutinin titer was negative. This post-infectious immune-mediated hemolysis has been increasingly recognized in severe babesiosis [13,14]. She required transfusion support, though immunosuppression was avoided, and her condition gradually improved as the infection resolved.

During hospitalization, serial laboratory evaluations demonstrated a steady decline in parasitemia from 9.7% to 0% by day 16. Lactate dehydrogenase (LDH) peaked at 1,573 U/L before normalizing, and the hemoglobin level reached a nadir of 6.5 g/dL. Haptoglobin was <10 mg/dL, consistent with ongoing hemolysis during the recovery phase. Total bilirubin peaked at 8 mg/dL, accompanied by mild, transient elevations in hepatic transaminases. Imaging studies revealed bilateral ground-glass opacities and small pleural effusions, while transthoracic echocardiography demonstrated preserved systolic function with a left ventricular ejection fraction of 68% [7,9,10].

## Discussion

This case demonstrates several important points. Asplenic patients are at particularly high risk for severe babesiosis, with high parasitemia and related complications. Therapeutic challenges may arise due to drug allergies, but careful reevaluation can allow for the use of guideline-based azithromycin-atovaquone therapy. Another key feature is the development of autoimmune hemolysis, as evidenced by worsening anemia despite parasite clearance and a complement C3-positive direct antiglobulin test, highlighting complement-mediated hemolysis as an emerging complication of babesiosis. Finally, optimal management required a multidisciplinary approach involving infectious disease, hematology, and transfusion medicine specialists.

Babesiosis is an emerging tick-borne parasitic infection in the United States, with *Babesia microti* being the most common species transmitted by *Ixodes scapularis* ticks in the Northeast and upper Midwest [2,8,9]. Clinical manifestations vary from asymptomatic infection to severe, life-threatening illness, particularly in asplenic, elderly, or immunocompromised patients. This case highlights several important clinical features and complications of babesiosis, including severe parasitemia, respiratory failure, and immune-mediated hemolysis.

Our patient’s history of splenectomy placed her at high risk for severe babesiosis. The spleen plays a critical role in clearing parasitized erythrocytes; asplenic patients often develop higher parasitemia and more severe complications [2,3,9,10]. Additionally, advanced age and comorbidities contribute to poor outcomes. First-line therapy for babesiosis typically includes azithromycin plus atovaquone for mild to moderate cases, while clindamycin plus quinine is reserved for severe illness or intolerance to macrolides [2,12]. Our patient was initially treated with clindamycin plus atovaquone due to a reported azithromycin allergy, though this regimen is less well established. Ultimately, a successful azithromycin challenge allowed transition to the standard regimen.

Literature recommends that for severe or prolonged babesiosis, especially in highly immunocompromised patients, antimicrobial therapy should be extended to at least six consecutive weeks, including at least two final weeks during which parasites are no longer detected on peripheral blood smear, instead of the standard 7-10 days [12].

In cases of severe babesiosis with parasitemia ≥10% or organ dysfunction, red blood cell exchange transfusion is recommended to rapidly reduce parasitemia and improve oxygen delivery [2,3,12]. In this patient, partial exchange transfusion effectively reduced parasitemia from 9.7 to 5.2%, aligning with guideline-based management. Clinicians should consider exchange transfusion not only for high-grade parasitemia (>10%) but also for patients with severe hemolytic anemia or pulmonary, renal, or hepatic compromise, regardless of parasitemia level, maintaining a low threshold for intervention [1,2]. The decision should be based on the overall clinical state and evidence of end-organ dysfunction rather than parasitemia alone.

The patient’s course was complicated by respiratory failure with bilateral ground-glass opacities and pleural effusions. Pulmonary involvement in babesiosis is uncommon but has been reported in severe cases, likely reflecting a combination of systemic inflammation, capillary leak, and high parasitic burden [4-6,13]. Pulmonary injury in babesiosis is likely multifactorial. Systemic inflammation and cytokine release in response to parasitic lysis can increase pulmonary capillary permeability, leading to non-cardiogenic pulmonary edema akin to acute respiratory distress syndrome (ARDS). Intra-vascular hemolysis releases hemoglobin, heme, and inflammatory mediators, which may induce oxidative endothelial injury within pulmonary microvasculature. Changes in red blood cell deformability or microvascular sludging, possibly augmented by parasite-induced erythrocyte membrane alterations, can impair microcirculatory flow in the lung. Finally, paradoxical worsening of lung injury has been described following initiation of antiparasitic therapy due to enhanced inflammatory response to dying parasites. These combined effects may precipitate alveolar flooding and hypoxemic respiratory failure [13].

Of particular interest was the development of complement-mediated hemolysis following parasite clearance. Hemolytic anemia in babesiosis may occur due to direct parasitic destruction of red cells or as a delayed immune-mediated process. Our patient demonstrated a positive complement (C3) but a negative IgG direct antiglobulin test (DAT), consistent with complement-driven hemolysis rather than classic warm autoimmune hemolytic anemia (WAHA) as the immune-mediated hemolytic process associated with babesiosis, particularly in asplenic or immunocompromised patients [14].

This case underscores several key teaching points as follows: high-risk populations (asplenic, elderly, immunocompromised) are more likely to develop severe babesiosis and require aggressive management. This case illustrates adherence to the current Infectious Diseases Society of America (IDSA) guidelines, while underscoring the importance of individualized, patient-centered management based on disease severity and clinical context [12]. Exchange transfusion remains a cornerstone of therapy in severe disease with high parasitemia. Immune-mediated hemolysis can complicate recovery despite parasite clearance and should be considered in patients with persistent anemia and hemolysis. Careful antibiotic selection and allergy evaluation are critical, as macrolide-based regimens are the preferred therapy. Severe babesiosis can manifest with multiorgan dysfunction and post-infectious immune-mediated complications, and may also exhibit a high recurrence rate in immunocompromised patients that can lead to further severe outcomes. Awareness of atypical features, such as complement-mediated hemolysis, is important for timely recognition and management. This case contributes to the growing body of literature on severe babesiosis and highlights the complexity of caring for high-risk patients.

## Conclusions

Severe babesiosis, particularly in asplenic patients, can present with high parasitemia, pulmonary complications, and complement-mediated hemolysis. Early recognition of high-risk patients is critical to prevent multiorgan involvement. Reported drug allergies should be carefully reassessed, as many are inaccurate; when appropriate, controlled allergy challenge testing can safely confirm or exclude true hypersensitivity, allowing use of optimal first-line antimicrobial therapy. Even after parasite clearance, close monitoring for immune- or complement-mediated hemolysis is essential to detect post-infectious complications. Multidisciplinary collaboration among infectious disease, hematology, and critical care teams remains central to individualized management and improved outcomes in severe babesiosis.
